# Public acceptability of government intervention to change health-related behaviours: a systematic review and narrative synthesis

**DOI:** 10.1186/1471-2458-13-756

**Published:** 2013-08-15

**Authors:** Stephanie Diepeveen, Tom Ling, Marc Suhrcke, Martin Roland, Theresa M Marteau

**Affiliations:** 1 RAND Europe, Cambridge, UK; 2 Norwich Medical School, University of East Anglia, Norwich, UK; 3 Behaviour and Health Research Unit, Institute of Public Health, University of Cambridge, Cambridge, UK

**Keywords:** Health behaviour, Attitude, Public opinion, Policy

## Abstract

**Background:**

Governments can intervene to change health-related behaviours using various measures but are sensitive to public attitudes towards such interventions. This review describes public attitudes towards a range of policy interventions aimed at changing tobacco and alcohol use, diet, and physical activity, and the extent to which these attitudes vary with characteristics of (a) the targeted behaviour (b) the intervention and (c) the respondents.

**Methods:**

We searched electronic databases and conducted a narrative synthesis of empirical studies that reported public attitudes in Europe, North America, Australia and New Zealand towards interventions relating to tobacco, alcohol, diet and physical activity. Two hundred studies met the inclusion criteria.

**Results:**

Over half the studies (105/200, 53%) were conducted in North America, with the most common interventions relating to tobacco control (110/200, 55%), followed by alcohol (42/200, 21%), diet-related interventions (18/200, 9%), interventions targeting both diet and physical activity (18/200, 9%), and physical activity alone (3/200, 2%). Most studies used survey-based methods (160/200, 80%), and only ten used experimental designs.

Acceptability varied as a function of: (a) the targeted behaviour, with more support observed for smoking-related interventions; (b) the type of intervention, with less intrusive interventions, those already implemented, and those targeting children and young people attracting most support; and (c) the characteristics of respondents, with support being highest in those not engaging in the targeted behaviour, and with women and older respondents being more likely to endorse more restrictive measures.

**Conclusions:**

Public acceptability of government interventions to change behaviour is greatest for the least intrusive interventions, which are often the least effective, and for interventions targeting the behaviour of others, rather than the respondent him or herself. Experimental studies are needed to assess how the presentation of the problem and the benefits of intervention might increase acceptability for those interventions which are more effective but currently less acceptable.

## Background

Much of the burden of disease worldwide, including cancers, cardiovascular disease and diabetes, could be reduced if people changed their behaviour, e.g. stopped smoking, reduced their alcohol intake, ate healthier diets and became more physically active. Policy makers have a variety of means at their disposal by which they can try to influence these behaviours ranging from the provision of information to the public, through to measures that restrict choice by regulation [1].

Increasingly policy makers are interested in how to approach changing behaviour in populations, but face a lack of clarity on how best to do so. This is exacerbated by the increasing recognition that traditional, information based interventions to change behaviour have had modest or no effects [2]. In choosing between interventions, evidence of effectiveness and cost are important considerations on which much research and professional activity has focused. Examples include the development of evidence synthesis methods by the Cochrane Collaboration and the US Agency for Healthcare Quality and Research as well as evidence-based guidelines from sources such as the Guide to Community Preventive Services in the USA [3] and NICE guidelines in the UK [4]. Such activity is aimed at ensuring that evidence of effectiveness and cost-effectiveness are captured in public health policies.

A further consideration for governments in deciding how to intervene to change behaviour is the attitude of the public towards such interventions, and the extent to which any interventions are likely to be acceptable. This matters, not only because levels of acceptability may critically affect the effectiveness of the intervention, but also because accountable governments need to be aware of public attitudes if they want to act in the public’s interest while at the same time maximising their own chances of being re-elected. There has been less focus on this as an area of academic study. A number of recent surveys suggest that attitudes vary with the nature of intervention, with the provision of information being more acceptable to the public than regulation to limit behaviours or restrict the use of particular products [5,6]. Beyond this general impression, we do not know the extent to which these attitudes vary across behavioural domains, how they relate to the nature of the intervention or to the populations surveyed, nor how attitudes vary with the ways in which the need for and consequences of interventions are framed, despite the relevance and interest of these considerations to policy.

In this review, we synthesise evidence on public attitudes towards government intervention in relation to four key sets of behaviours: alcohol consumption, smoking, diet and physical activity. We focus on these four sets of behaviour given their significant contribution to preventable premature morbidity and mortality [1]. We examine the evidence for how the acceptability of interventions varies with the characteristics of the target behaviour, the type of intervention and the respondents. In addition we examine the extent to which any variations are contingent on the framing of the problem or the intervention.

## Methods

We conducted a scoping review to identify and summarise relevant literatures on factors which influence public acceptability of public health policies designed to change behaviour. Scoping reviews are reviews that map or describe rather than evaluate an area of literature in which there are considerable uncertainties about its nature or parameters. We synthesised the data extracted narratively, given the heterogeneity of the study methods and data extracted. We included studies that involved measurement of attitudes towards policies involving interventions to change behaviours to reduce smoking, alcohol and food consumption and to increase physical activity. Studies were located using the following databases: Econlit, Academic Search Elite, Business Source Plus, ERIC, Social Sciences Abstracts, Web of Science, Science Direct and Google Scholar. The search was conducted using a range of behaviour-related keywords (e.g. exercise, alcohol, smoking, diet) used in combination with policy (e.g. regulation, law, intervention) and attitude-related keywords (e.g. opinion, perspective). The search strategy included studies published in English between 1980 and April 2011. Bibliographies of included studies were also reviewed to retrieve any further references. Pilot searches indicated that very few references came from countries outside Europe, Australia, New Zealand, Canada and the United States and we therefore restricted our search to work carried out in these countries.

We reviewed titles, abstracts and full texts. We only included studies that examined the general population or a subset of the population (e.g. particular age, gender or occupation groups), and directly analysed their attitudes towards the acceptability of an intervention or policy. We therefore excluded studies solely looking at views of policy makers, and we also excluded studies where there was a clear vested interest, e.g. the opinion of industry groups on increasing alcohol taxation.

Data were extracted on study design, sample, data collection and analysis methods, and findings. To summarise the evidence base and its shortcomings, we first mapped the studies across policy domains, countries, and methods used. We then analysed the nature and scope of the evidence on acceptability and the potential effect of three key areas of policy interest: characteristics of (a) the target behaviour, (b) the intervention, and (c) the respondents. There were some cases where time series survey data were used and later articles reported cumulative and comparative findings across the years. In these cases, we reference findings from the most recent publication. Full details of the search strategy, search terms, methods of synthesis, and a PRISMA flow diagram are obtainable from the corresponding author.

## Results

The initial search produced 6,979 records from database searches plus additional references from organisational websites and snowballing. After screening titles, 1,654 abstracts were reviewed. This generated 213 empirical papers for more detailed analysis, representing 200 unique studies eligible for inclusion in the review as some studies were represented in more than one paper.

We first describe the characteristics of studies which were included.

### Characterisation of the studies

Over half the studies investigated attitudes to speculative or impending policy conditions (118/200, 59%) with thirty-eight per cent (76/200) examining attitudes towards an existing policy. Just ten of the 200 studies collected data as part of experimental designs, of which four were multi-criterion mapping studies and six were controlled trials [7-17]. Of the 190 non-experimental studies, most (162/190, 84%) drew on data from surveys and opinion polls. Most of the survey-based studies (112/162, 69%) reported statistical associations between a range of factors and the acceptability of policies, with the remaining 50 providing a narrative analysis of survey results. Of the qualitative studies identified (28/200, 13%), the majority drew on data from interviews (13/28), the rest on focus groups (6/28), a mixture of focus groups and interviews (5/28), interviews and field observations (2/28) or textual analyses or analyses of secondary data.

#### Distribution of studies by country

The largest number of studies was conducted in the USA (91/200, 46%). Only a few studies (15/200, 8%) used a comparative European or international sampling frame (Table 1).

**Table 1:** Types of interventions presented across studies by behavioural domain

**Level of intrusiveness**	**Total**	**Alcohol**	**Smoking**	**Smoking and alcohol/Diet**	**Diet**	**Physical activity**	**Diet and physical activity**
Providing information	30 (10%)	9 (12%)	7 (5%)	2 (11%)	1 (5%)	1 (33%)	10 (25%)
Guiding choice by providing incentives or disincentives	76 (26%)	32 (43%)	21 (16)	7 (39%)	3 (16%)	0 (0%)	13 (33%)
Restricting or eliminating choice	182 (64%)	33 (44%)	106 (79%)	9 (50%)	15 (79%)	2 (67%)	17 (43%)
Total	288 (100%)	74 (100%)	134 (100%)	18 (100%)	19 (100%)	3 (100%)	40 (100%)

### Acceptability of policies to change behaviour

In what follows we report on the main patterns and characteristics identified across the studies. We present the results in terms of the characteristics of the behaviours, the interventions and the respondents.

#### Characteristics of behaviours included in the review

The behaviours most commonly studied related to smoking (110/200; 55%), followed by alcohol (42/200; 21%), diet (18/200; 9%), combined diet and physical activity-related behaviours (18/200; 9%), with fewest concerning physical activity alone (3/200; 2%).

Of the 105 studies measuring absolute support for specific policy options (rather than relative support for different policy options), 99 studies reported majority support for some form of intervention to change behaviour, regardless of the behavioural domain. For smoking there was consistent support for some form of restriction on smoking-related behaviours, and all of the studies that looked at changes in the acceptability of smoking restrictions over time found that support increased with time. Consensus support was also found for interventions that in some way restricted diet and behaviours related to physical activity, with no studies reporting a majority opposed to these types of intervention. There was slightly more variation in support for alcohol control policies, particularly over time: three studies reported a decline in support for more intrusive interventions over time [18-20].

#### Characteristics of interventions included in the review

Two characteristics of the interventions were considered in terms of how they might influence the acceptability of interventions: the degree of intrusiveness; and, the stage of implementation of a policy designed to change behaviour. We also considered how acceptability might have varied with the framing of the intervention.

##### Intrusiveness of the intervention

Interventions can be classified according to the degrees of intrusiveness they involve, as in the ‘Nuffield intervention ladder’ for which intrusiveness was considered relative to individual freedom and responsibility, as involving state intervention [1]. We classified the interventions presented in each study into one of three groups based on the Nuffield Intervention Ladder: Providing information; Guiding choice; Restricting or limiting choice (Table 2). In total, 288 types of interventions, distinguished by level of intrusiveness, were discernible. The majority of these involved restricting or eliminating choice (64%), with fewer involving the use of incentives to guide choices (26%), with the smallest proportion involving the provision of information (10%). This pattern was evident across all behavioural domains.

**Table 2:** Country and behavioural domain of included studies

	**Total**	**Alcohol**	**Smoking**	**Smoking and alcohol/diet**	**Diet**	**Physical activity**	**Diet and physical activity**
United States	91	20	52	2	8	1	8
Canada	14	6	8	0	0	0	0
Australia or New Zealand	37	7	20	3	5	0	2
United Kingdom	15	2	7	2	2	0	2
Other European	25	5	14	0	1	2	3
Central Asia and Eastern Europe (Russia or Armenia)	3	0	3	0	0	0	0
Comparative: within the European Union	9	1	3	0	2	0	3
Comparative: more than one of Canada, United States, Great Britain, Australia and New Zealand	6	1	3	2	0	0	0
Total	200	42	110	9	18	3	18

Examples of interventions that involved restricting or eliminating choice were, for tobacco, mandatory plain packaging for tobacco products, and for tobacco and alcohol, restrictions on advertising, limiting the number and venues for sale, and age restrictions on consumption or purchasing. Examples of information provision across all behavioural domains are information campaigns and, for diet, tobacco and alcohol, the use of various product labels. The final set of interventions focused on encouraging or discouraging behaviours by providing incentives, disincentives or both. These included taxation, subsidies, and penalties for social harms associated with unhealthier behaviours (e.g. drink driving).

Support was generally higher for interventions perceived as less intrusive. For example, warning labels and educational campaigns were consistently more likely to be supported than policies introducing disincentives designed to influence behaviour such as tax based incentives to discourage smoking or excessive alcohol consumption. This finding was evident across behavioural domains [5,9,10,12,18,19,21-26]. Respondents were also more likely to support intrusive measures aimed at commercial businesses than interventions aimed at individuals [5]. Support for intrusive interventions was generally strongest for tobacco control. This was most marked for smoking bans in workplaces, and indoor public places including restaurants and shopping centres [27-30], with more variation in the level of support for bans in bars, pubs and outdoor places [27,31-37]. For alcohol, there was strong support for education interventions and for increasing penalties for drink-driving [20]. The majority of studies (17/21) suggested that there was little support for alcohol price-related policies and only a small number of studies (5/21) showed majority support for limits on the sale of alcohol products, such as limiting sale to drunken persons [38], limiting store hours [39], or limiting sale at corner stores [19,20,22]. However in some cases, there was support for specific pricing policies for example to increase alcohol taxes with the aim of reducing underage drinking [40], to deal with problems from alcohol use [41], or to lower taxes on low alcohol beverages [41]. The presentation of the intervention was important, with more support generally expressed for measures aimed at reducing alcohol consumption among those underage, or in particular locations such as sporting events, licensed premises (by restricting licences) and college campuses, compared with policies to restrict happy hours, increase the price of alcohol, reduce the number of sales outlets or the trading hours of pubs and clubs, or increase the minimum drinking age [31,40,42].

There was generally strong support for policies that focused on changing the behaviour of children and young people [6,43-47]. For example, respondents were more supportive of restrictions on tobacco and alcohol sales to minors [8] as compared to policies that restricted availability (e.g. opening hours of retail outlets) [48], involved sanctions against adult consumers [23,49] or banned advertisements [50]. Bans on smoking in cars with children present also attracted strong support. In diet and physical activity-related behaviours, attention was focused on interventions in schools, as a key area to influence childhood behaviour [6,51-54] School-based interventions were the focus for twelve studies looking at both diet- and physical activity-related interventions (12/18, 67%) and seven studies solely examining diet-related interventions (7/18, 39%). Of these studies, fourteen indicated strong support for school-based interventions. Only one study that measured general support for eliminating junk food in schools found less than majority support [55].

##### Stage of policy implementation

Support for policies was associated with the stage of implementation, with interventions generally becoming more acceptable once they had been introduced. For example, support for smoking bans increased following the introduction a smoking ban [56-61] with the change more pronounced for smokers than non-smokers or ex-smokers [61-65]. However, in the case of alcohol, support for alcohol control policies appeared in some cases to erode over time, judged by serial cross-sectional surveys from Canada, the US, and Australia [18-20]. However, looking at these surveys in more detail suggests that some types of interventions had sustained high levels of support over time: even though there was a general decline in support for intrusive measures, there was no decline in support for banning alcohol advertising on TV or for banning alcohol sponsorship at events. In addition, while overall support declined, the rank order of support between specific policies remained largely unchanged [19,20].

##### Framing of the intervention

Only one experimental study directly examined how framing of an intervention might influence its acceptability [15]. In this case an education intervention was more acceptable when responsible drinking messages were framed in personalised terms rather than group norms.

### Characteristics of respondents

Explanations for variation in responses to surveys focused largely upon characteristics of respondents, with gender, age and respondents’ salient health-related behaviours frequently considered. We also report on how acceptability was patterned by socioeconomic status and country of participants.

#### Gender

The responses of men and women were examined separately in the majority of tobacco-related studies, about half of alcohol studies but fewer than a fifth of studies on diet and physical activity. Gender was strongly associated with levels of support for policy interventions across behavioural domains, with the strongest associations for interventions to influence smoking-related behaviours.

Consistently, studies reported that women had higher levels of support for a range of alcohol control policies compared to men (21/26, 81%) [15,18-20,22,38,39,42,48,66-78] and tobacco (22/32, 69%) [27,35,50,73,79-96], though three studies found higher levels of support among men for specific policies, including regulation of the tobacco industry [97], plain packaging [98], and bar workers for smoke-free workplaces [60]. These differences raise questions about the reasons why gender might impact on levels of support, and its relevance relative to other variables. Several studies suggest that the type of intervention might affect the extent to which gender affects acceptability [8,35,38,42,66,70,99]. While women were generally more supportive of policies, there were particular cases in which studies noted no significant difference between men and women, specifically hospital bans [35,99], punitive lawsuits for traffic-related injuries [66], a minimum legal age of consumption and surveillance of restaurants and retail outlets [38]. However, the reasons why gender might affect levels of support remain largely unexplored. One possibility is that women were less likely to indulge in these behaviours which we find (see below) was a factor which influenced acceptability, but this was not generally explored by the authors of these studies specifically in relation to gender.

Fewer studies reported on differences between men and women in support for diet and physical activity-related interventions. In these, women were generally more supportive of obesity-oriented interventions than were men [21,43,44,55] as they were of restrictions to limit access to higher fat foods and drinks [54].

#### Age

Older respondents were generally more supportive of restrictive measures around alcohol [20,38,48,80], smoking [100], and diet [26,44,54,55,101].

#### Socioeconomic status

Twenty studies examined how attitudes to policies might differ by socioeconomic status. Most often income was used as a sole measure of socioeconomic status. Findings were most consistent for smoking-related behaviours, for which five out of six studies suggested higher income groups were more supportive of intrusive interventions. However, there were few strong trends, with over half of studies finding only small or modest associations between attitudes and two studies finding no effect. The association between socio-economic status and attitudes to alcohol control policies was also generally small, with different studies finding that either lower or higher income groups could be more supportive of interventions [19,42,67,77]. The picture was even more ambiguous for diet and physical activity-related policies, with two out of seven studies finding lower income groups to be both more supportive of interventions [55,102] while three found that they were less supportive compared to higher income groups [21] with no effect in one other study [103]. Studies looking across behaviours suggested a complex picture, with variation between country- and individual- level wealth and support for different policies, including variation in associations between behaviours [5,6].

#### Country

Seven out of ten studies comparing responses across countries showed substantial differences in support for policies to change behaviour between countries, with differences apparent in studies using a range of methods [5,10,12,13,70,90,104]. Support for restrictive policies in the areas of smoking and diet was generally higher in more authoritarian countries than liberal democracies with, for example, 88% support in China for partially restrictive interventions or those making the behaviour more expensive, compared to 46% in the USA [5].

#### Respondents’ own behaviour, health and experience

In most studies in which it was considered, acceptability varied with respondents’ own health-related behaviour. For example, non-smokers and ex-smokers were more likely to support tobacco control interventions than smokers [23,24,29,34-36,47,49,80,85],[87,88,92,93,96,99,105-111]. Likewise in a majority of studies, respondents who regularly consumed alcohol were less likely to support intrusive alcohol-related interventions [18,20,22,38,39,69,71,76],[112,113]. There were some exceptions in the area of alcohol control policies with one study suggesting that heavy drinkers were just as supportive of restrictive policies as moderate drinkers [19,68]. Few studies assessed the association between acceptability and an individual’s own diet and physical activity. Of the six that did, four found no significant association. Of the two studies indicating a significant association, one study reported that those who exercised more were more supportive of policies to address obesity, also finding those with high BMI were more likely to support regulation of advertisements and school-based fast food concessions [55]. However, in the other study those with high BMI had less positive attitudes towards the use of food labels to reduce obesity [101].

An individual’s awareness and experience of harm from the target behaviour may also influence acceptability. We found eleven studies that considered this. Regarding personal experience of harm, all four studies reported an association between personal experience and support for restrictive policies. For example, in the area of alcohol and smoking, support for restrictive policies was highest amongst those reporting experience of a death related to the behaviour [114] or experience of harm relating to the behaviour [115,116]. In relation to awareness of harm, six out of eight studies found that respondents who were more aware of the harms associated with a behaviour were more likely to support policies to restrict that behaviour. For example, knowledge of harms increased support for policies designed to restrict smoking and second-hand smoke [55,77,90,95,110,117,118].

## Discussion

### Summary of main findings

Acceptability of interventions to change behaviour varied across behavioural domains (with interventions on smoking attracting more support), as a function of the type of intervention (less intrusive interventions attracting more support), whether the intervention had already been implemented (greater support being reported for interventions already implemented), and the target of the intervention (interventions targeting children and young people generally more strongly supported). Acceptability also varied with the characteristics of the respondents (those engaging in the targeted behaviour being less supportive of interventions to stop the behaviour than others, and women and older respondents more likely to endorse more restrictive measures). We found only one study assessing the impact of the framing of survey questions upon acceptance.

### Discussion of main findings

(a) Behaviour

There are several possible reasons why support for tobacco control interventions was generally high and higher than for interventions for other behavioural domains. First, the majority of people in high income countries no longer smoke [119], so stronger support for tobacco control policies than, say, alcohol policies may reflect a preference for interventions that affect the behaviour of others. Second, there is a high level of awareness of the harm from tobacco, amongst smokers and non-smokers, which may lead to more support for interventions designed to reduce this harm. Third, there is a strong recent history for tobacco control interventions in the form of taxation increases and restrictions on where tobacco can be smoked and purchased. Public attitudes change with time and appear to often be influenced by the enacting of legislation, as shown in several studies by the increased acceptability of restrictions on smoking after the introduction of bans on smoking in public places [58,60]. This may be explained by a process of cognitive dissonance whereby attitudes follow behaviour (rather than vice versa, which is the more commonly assumed route to behaviour change) [120], or by the operation of the status quo bias, a preference for the current state of affairs [120,121].

(b) Intrusiveness of intervention

The greater acceptability of less intrusive interventions is illustrated in one poll in which 82% of those surveyed supported drink labelling (an intervention with no evidence of effectiveness in reducing alcohol consumption) compared with 45% who supported the setting of a minimum price per unit of alcohol (an intervention for which there is good evidence for effectiveness in reducing alcohol consumption) [6]. Generally more restrictive policies are more effective, although not always. This finding appears consistent with the traditional economic world-view that people know best themselves what is good for them and are thus reluctant to accept public policy intervention that significantly interferes with their own decisions. Instead they tend to prefer interventions that do at best indirectly affect them (e.g. public awareness campaigns, education). The finding raises two questions: can public attitudes towards more effective interventions be changed and if not, when are governments justified in intervening regardless of public opinion?

Literatures from social psychology and moral psychology suggest two broad approaches to changing attitudes: first, targeting the beliefs that underlie attitudes; and second, activating the core values upon which acceptability judgments are based. Regarding the first of these approaches, information provided about the harmfulness of the target behaviour and the effectiveness of the proposed intervention to reduce this harm would be predicted to alter attitudes towards intervention [122]. In keeping with this, a series of experimental studies found that the acceptability of financial incentives for stopping smoking and sustaining weight loss increased with rising effectiveness [123]. Perceptions of the cause of unhealthy behaviour (e.g. individual decisions versus those shaped by the environment) also influence the perceived effectiveness and acceptability of interventions [44,124]. The second approach is based on observations in moral psychology that judgments are influenced by a series of core values (e.g. fairness). From this it would be predicted that acceptability would be increased by framing the outcomes of different interventions in terms of a core moral value such as fairness to those who are the focus of the intervention (e.g. children who are the focus of obesity prevention programmes) or those who are not the focus but who stand to gain by a change in behaviour (e.g. tax payers whose contribution towards the adverse health and criminal consequences of excessive alcohol consumption would be lessened by effective interventions). Framing consequences of interventions to target both beliefs and core values is likely to have the largest impact on acceptability.

Regarding when governments might be justified in intervening regardless of public opinion, one economic justification is when there is a ‘market failure’, *i.e.* when the market, if left unchecked, produces a sub-optimal outcome from a societal perspective [125]. Potential market failures relevant in the current context are imperfect information (e.g. people under-estimating the harm caused by certain health-related behaviours, in particular if those consequences materialise only far ahead in the future), and externalities (e.g. the costs and consequences borne by second-hand smoking). An additional justification might arise if the people are acting with “bounded rationality”, rather than in the well-informed way that traditional economic theory assumes. This approach, largely subsumed under the heading of Behavioural Economics, offers a broadened set of rationales for when governments may be justified to interfere with individual decisions in the area of health behaviours [125].

(c) Characteristics of respondents

Acceptability varied with the three characteristics of respondents that we focused upon: gender, age and whether or not the individual engaged in the behaviour that was the target of the intervention.

The finding that women are more likely to support intrusive (and often more effective) interventions is intriguing. Given women in OECD countries behave more healthily than men, at least in terms of tobacco and alcohol consumption (which is what the bulk of the reviewed studies focused on), this finding may again reflect our earlier observation that people are less resistant to interventions that target the behaviour of others. Women’s preference for stronger measures may also reflect more direct experience of the adverse consequences of certain unhealthy behaviours (e.g. from alcohol-related harm experienced by and coming from their male partners). In addition, women more frequently provide informal care than do men [126], perhaps leading to a stronger preference for interventions that prevent the need for care.

We are uncertain why the acceptability of intrusive interventions increases with age. This may reflect a greater awareness of the burden of disease with age. Alternatively it may reflect a growth in trust of government intervention with age. Given older individuals are more likely to vote, the patterning of acceptability by age is likely to be of particular interest to politicians.

The finding that people engaging in unhealthier behaviours are more likely to reject policies that aim to restrict such behaviours is consistent with the world-view that individual preferences for public interventions are determined by people’s self-interest. (The self-interest “theory” has also been invoked in the study of the determinants of public support for other types of public policies, e.g. taxation or the welfare state [127]. This may mean that either those individuals take the rational, utility-maximising decision to engage in, say, smoking and thus consider any external interference as utility-decreasing; or while the unhealthy behaviour results from the individual pursuit of self-interest, the individual may not have been in a position to carefully weigh the costs and benefits of its actions to take a rational enough decision.

Conversely, those not engaging in the unhealthy behaviour that is the focus of intervention appear more willing to advocate interventions that restrict the behaviour of others. That may again be compatible with the self-interest interpretation, if, say, the non-smokers advocate intrusive and effective interventions because they experience the adverse external effects (e.g. in terms of second-hand smoking) resulting from the behaviour and so want to reduce those.

### Strengths and limitations of the current review

The strength of this review is its novelty, being the first systematic attempt to map the evidence on public acceptability of government interventions to change health-related behaviour. We have identified emerging themes and gaps in the research evidence and ways in which future studies can be strengthened. The review was limited in several ways. While the search was systematic, in keeping with the scoping nature of the review, we did not use formal methods to synthesise the results. The literature was heterogeneous in several salient dimensions. Different questions and response options were provided, which influenced responses [37,128]. Different data-collecting methods were used, which also influenced the attitudes expressed [129]. The respondents varied in age, gender, socio-economic status, ethnicity and country of residence, and different methods were applied across countries in order to account for those factors in determining public attitudes. The context of the surveys varied in time, source of funding, political and policy contexts, all of which are likely to shape attitudes but not always in a way that was revealed in the individual studies or in our narrative synthesis. Of particular note is the provenance of the studies with the majority from the USA. Future reviews that focus on particular behaviours or types of interventions, or that select designs from a narrower range can allow some of these potential influences on public acceptability to be teased out.

### Implications for policy makers

Policy makers are sometimes faced with lack of support for interventions that have the greatest chance of achieving policy objectives. For example, a recent WHO review of alcohol control policies noted that the most effective interventions (pricing and availability) are those for which the public is most sceptical [130]. While the authors of this report call for research on increasing public and political support for the most effective interventions, this is more an aim of lobbyists. The research question is better framed in terms of whether intervention effectiveness and public acceptability can be more closely aligned and if so how. The current review identified an evident gap in the research literature to inform this question, which we consider in the section below.

### Implications for research on public attitudes

Revealing the determinants of public preferences towards behaviour change policies is a challenging research endeavour. Based on the existing evidence we reviewed, there is considerable scope for more research in this complex and highly policy relevant domain. Existing literature on public attitudes towards interventions to change health-related behaviours seems largely uninfluenced by the large literatures on judgment and decision-making [131] and moral judgments [132]. By omitting reference to pertinent social and behavioural science literatures on judgment, policy-makers and others are provided with a partial view of public opinions and of the options available to policy makers to influence acceptability. Its inclusion will provide a more valid account of public acceptability of the more intrusive interventions, which often have the most potential to change behaviour to improve population health. It will also provide the theoretical basis for intervening to assess the extent to which public opinions are conditional upon information provided about the likely impact of interventions. Finally, it will also allow for an exploration of how framing of interventions, to align with a population’s dominant core values and beliefs, could be a tool for influencing acceptability.

The stability of public attitudes towards interventions to change health-related behaviour is little studied. When governments plan regulation that has the potential to reduce sales, campaigns are invariably launched by the affected companies including alcohol, tobacco and food and beverage manufacturers [19,133]. The malleability of public opinion by competing messages from governments and commercial companies warrants investigation.

There is particular scope for adding to the literature in the domains of physical activity and diet, where we found very few studies. From a methodological viewpoint, while we see potential for a more extensive analysis of existing, preferably international survey data, we see particular promise in the development of discrete choice experiments that – in contrast to most opinion poll data – confront respondents with trade-offs between different policy options, providing a more valid way of assessing acceptability.

## Conclusions

Public acceptability of government interventions to change behaviour is greatest for the least intrusive interventions, which are often the least effective, and for interventions targeting the behaviour of others, rather than the respondent him or herself. Experimental studies are needed to assess how the presentation of the problem and the benefits of the intervention might increase acceptability for those interventions which are more effective but currently less acceptable.

## Competing interests

The authors declare that they have no competing interests.

## Authors’ contributions

TMM and MR had the original idea for the review The search strategy was developed by SD and TL, in discussion with TM and MR. The search, data extraction and synthesis was conducted primarily by SD in close collaboration with TL. The first draft of the paper was prepared by SD, with major contributions to further drafting from all authors. All authors read and approved the final manuscript.

## Pre-publication history

The pre-publication history for this paper can be accessed here:

http://www.biomedcentral.com/1471-2458/13/756/prepub
